# Gamma-delta T-cell large granular lymphocytic leukemia in the setting of rheumatologic diseases

**DOI:** 10.3389/fcell.2024.1434676

**Published:** 2024-08-01

**Authors:** Vadim Gorodetskiy, Yulia Sidorova, Bella Biderman, Natalia Kupryshina, Natalya Ryzhikova, Andrey Sudarikov

**Affiliations:** ^1^ V.A. Nasonova Research Institute of Rheumatology, Moscow, Russia; ^2^ Laboratory of Molecular Hematology, National Medical Research Center for Hematology, Moscow, Russia; ^3^ Hematopoiesis Immunology Laboratory, Russian Cancer Research Center N.N. Blokhin, Moscow, Russia

**Keywords:** gamma-delta T-cell, large granular lymphocytic leukemia, rheumatologic diseases, STAT3 mutation, STAT5B mutation

## Abstract

**Background:**

T-cell leukemia originating from large granular lymphocytes (T-LGL leukemia) is a rare lymphoid neoplasia characterized by clonal proliferation of large granular T lymphocytes expressing αβ or γδ T-cell receptor (TCR) on the cell membrane. γδT-LGL leukemia, accounting for approximately 17% of all T-LGL leukemia cases, is associated with autoimmune diseases. However, the features of γδT-LGL leukemia in patients with rheumatologic diseases are still insufficiently characterized.

**Methods:**

In this retrospective study, 15 patients with rheumatologic disease-associated γδT-LGL leukemia were included. The patients were obtained from a single center from 2008 to 2023. Data related to clinical characteristics and rheumatologic diagnoses were collected. Immunophenotype evaluations as well as T-lymphocyte clonality (based on *TCR-γ*, *TCR-β*, and *TCR-δ* gene rearrangements), and signal transducer and activator of transcription (*STAT*) three and *STAT5B* mutation analyses (by next-generation sequencing) were performed on blood, bone marrow, and spleen samples.

**Results:**

All but one patient had rheumatoid arthritis (RA). In 36% of patients, manifestations of γδT-LGL leukemia were present before or concurrently with clinical manifestations of RA. Splenomegaly was observed in 60% of patients and neutropenia (<1.5 × 10^9^/L) was detected in 93% of cases. CD4^−^/CD8^−^ and CD4^−^/CD8^+^ subtypes were detected in seven cases each. Mutations in *STAT3* were detected in 80% of patients; however, *STAT5B* mutations were not detected. Evaluations of T-cell clonality and variant allele frequencies at *STAT3* in the blood, bone marrow, and spleen tissue revealed an unusual variant of CD4^−^/CD8^−^ γδT-LGL leukemia with predominant involvement of the spleen, involvement of the bone marrow to a less extent, and no tumor cells in peripheral blood.

**Conclusion:**

The mechanism by which γδT-LGL leukemia may induce the development of RA in some patients requires further investigation. Cases of RA-associated γδT-LGL leukemia with neutropenia and splenomegaly but no detectable tumor-associated lymphocytes in peripheral blood (the so-called splenic variant of T-LGL leukemia) are difficult to diagnose and may be misdiagnosed as Felty syndrome or hepatosplenic T-cell lymphoma.

## 1 Introduction

Normal T cells can be categorized into two major subsets based on the surface expression of alpha-beta (αβ) or gamma-delta (γδ) T-cell receptor (TCR) (Ahmad et al., 2005). Large granular lymphocytic (LGL) leukemia is characterized by the clonal expansion of lymphocytes with abundant cytoplasm containing fine or coarse azurophilic granules and a reniform or round nucleus with mature chromatin (Lamy and Loughran, 2003). Approximately 85% of LGL leukemia is of T-cell origin (T-LGL leukemia), while the remaining cases are represented by NK-cell tumors (Lamy and Loughran, 2003).

Characteristic clinical features of T-LGL leukemia are cytopenia (most commonly neutropenia), splenomegaly, and an increase in LGLs in the peripheral blood (Lamy and Loughran, 2003; Chan et al., 2017; Cheon et al., 2020; Drillet et al., 2023). Mutations in the signal transducer and activator of transcription (*STAT*) *three* gene are molecular markers of T-LGL leukemia and have been identified in 27–72% of cases (Jerez et al., 2012; Koskela et al., 2012; Fasan et al., 2013; Shi et al., 2018). Mutations in the *STAT5B* gene have been found in 2% of patients with LGL leukemia (Rajala et al., 2013). Mutations are typically found in exons 19–21 of the *STAT3* gene and exon 16 of the *STAT5B* gene (Teramo et al., 2020).

Similar to the postulated normal counterpart, Т cells in T-LGL leukemia express either αβTCR or γδTCR. In general, the γδ variant of T-LGL (γδT-LGL) leukemia has similar clinical features to those of the αβ variant (αβT-LGL) (Sandberg et al., 2006; Bourgault-Rouxel et al., 2008). However, γδT-LGL leukemia cases tend to be more symptomatic disease and have poorer outcomes compared with those with αβT-LGL leukemia (Barilà et al., 2023).

The association of T-LGL leukemia with various autoimmune diseases is a characteristic feature of this pathology (Lamy and Loughran, 2003; Couette et al., 2022; Drillet et al., 2023). However, the features of γδT-LGL leukemia in patients with rheumatologic diseases are still poorly studied. The aim of this study was to characterize patients with γδT-LGL leukemia in the setting of rheumatologic diseases. Here, we report clinical findings, immunophenotypic data, *TCR-γ*, -*β,* and -*δ* gene rearrangements, and *STAT3* and *STAT5B* mutation frequencies for a series of 15 patients with γδT-LGL leukemia associated with rheumatologic diseases.

## 2 Patients and methods

### 2.1 Data sources

This retrospective study included 15 patients over 18 years of age diagnosed with γδT-LGL leukemia and rheumatologic disease who were admitted to the V.A. Nasonova Research Institute of Rheumatology from 2008 to 2023. The following data were collected from patients: age, sex, blood count, LGL count, splenomegaly, and rheumatologic diagnosis.

### 2.2 Ethics statement and consent to participate

This study was approved by the Ethics Committee of the V.A. Nasonova Research Institute of Rheumatology (protocol #14 on the 16-11-2023). All patients gave written consent for the collection and analysis of specimens and for publication of their data.

### 2.3 Evaluation of T-cell clonality

T-cell clonality was examined using genomic DNA extracted from blood (14 cases), bone marrow (5 cases), and spleen tissue samples (4 cases). T-cell clonality based on the rearrangements of the *TCR-γ (Vγ-Jγ)*, *TCR-β (Vβ-Jβ, Dβ-Jβ),* and *TCR-δ (Vδ-Dδ-Jδ)* was evaluated in all cases. T-cell clonality assays were performed according to the BIOMED-2 standardized protocol (van Dongen et al., 2003). Polymerase chain reaction amplification was carried out using an automated DNA Engine Thermocycler (BioRad, Hercules, CA, United States), and fragments were detected using an ABI PRISM 3130 Genetic Analyzer (Applied Biosystems, Foster City, CA, United States). Data were analyzed using GeneMapper version 4.0 (Applied Biosystems).

### 2.4 Flow cytometric analysis

A four-color flow cytometric analysis was performed on peripheral blood (12 cases), bone marrow (3 cases), and spleen (1 case) specimens. Lymphocytes were gated using CD45 *versus* side-scatter dot-plots. Cells were stained with a panel of fluorescence-labeled monoclonal antibodies, including CD3, CD4, CD5, CD7, CD8, CD16, CD19, CD57, TCR-αβ, and TCR-γδ. Flow cytometry was performed using a BD FACSCanto™ II (Becton Dickinson, San Jose, CA, United States) system and FCS Express version 3 (*De Novo* Software, Los Angeles, CA, United States).

### 2.5 Immunohistochemical studies

Immunohistochemical studies were performed on four spleen and two bone marrow formalin-fixed paraffin-embedded tissue samples. The following antibodies were used at the dilutions recommended by the manufacturers: CD3 (polyclonal, Dako, Carpinteria, CA, United States), CD4 (clone 4B12, Dako), CD8 (clone C8/144B, Dako), CD16 (clone 2H7, Novocastra Laboratories, Newcastle upon Tyne, UK), CD20 (clone L26, Dako), CD43 (clone DF-T1, Dako), T cell restricted intracellular antigen 1 (TIA-1) (clone 2G9, Immunotech, Marseille, France), granzyme B (clone GrB-7, Dako), TCR-beta F1 (clone 8A3, Thermo Scientific, Waltham, MA, United States), TCR-gamma (clone γ3.20, Thermo Scientific). After dewaxing and heat-induced antigen retrieval, immunostaining was performed using an Autostainer Link 48 (Dako, Denmark) according to the manufacturer’s instructions. All immunostained samples were counter-stained with hematoxylin.

### 2.6 Evaluation of *STAT3* and *STAT5B* mutations by next-generation sequencing

*STAT3* mutations were examined using genomic DNA extracted from specimens of peripheral blood (14 patients), bone marrow (5 patients), and the spleen (4 patients). *STAT5B* mutations were examined using genomic DNA extracted from specimens of peripheral blood (13 patients), bone marrow (5 patients), and the spleen (3 patients).

Mutations in exons 19–21 of the *STAT3* gene and in exon 16 of the *STAT5B* gene were identified by next-generation sequencing. Appropriate DNA regions were amplified using primers for exons 19–20 and exon 21 of the *STAT3* gene, as described previously (Koskela et al., 2012). Primers 5′-TGT​TGG​GGG​GTT​TTA​AGA​TTT​CCT-3′ and 5′-TCA​GAA​TGC​GAA​CAT​TGT​TAC​CA-3′ were used to amplify exon 16 (product length 267 bp) of the *STAT5B* gene.

Sequencing libraries were prepared from amplified DNA fragments using Nextera XT DNA Library Prep and Nextera XT Index Kit v2 (Illumina, San Diego, CA, United States) according to the manufacturer’s instructions. Sequencing was performed on the MiSeq apparatus (Illumina) using MiSeq Reagents Kit v2–300 cycles (Illumina). Data processing, including filtering, removal of accessory sequences, mapping of reads, and searching for variants, was performed using open source Trimmomatic, BWA, SAMtools, and VarDict software (Li et al., 2009; Li and Durbin, 2010; Bolger et al., 2014; Lai et al., 2016). The variant allele frequency (VAF) threshold was set to 0.5%. For each target, 2000–5,000 reads were generally obtained. Cases with VAF less than 1.5% were considered positive, provided that the mutation was observed in a repeated analysis of the sample and/or in another sample from the same patient. Discovered variants were annotated according to COSMIC, ClinVar, refGene, and snp138 open databases using ANNOVAR software (Sherry et al., 2001; Wang et al., 2010; O'Leary et al., 2016; Landrum et al., 2018; Tate et al., 2019).

### 2.7 Statistical analysis

Descriptive statistics are presented as numbers and percentages for categorical data and as medians and ranges for continuous data. Overall survival was estimated by the Kaplan-Meier method and was calculated from the date of manifestation of T-LGL leukemia to death for any cause or last known follow-up for censored patients.

## 3 Results

The clinical and laboratory characteristics of the 15 patients included in the analysis are shown in Table 1. The patients were Caucasian and predominantly female (female: male of 1.5:1). The median age of the patients at the time of key T-LGL leukemia manifestations, such as neutropenia, lymphocytosis, or splenomegaly, was 58 years (range, 39–76 years). Fourteen patients had rheumatoid arthritis (RA) and one had primary catastrophic antiphospholipid syndrome (CAPS). Two patients had Sjögren syndrome combined with RA. Additionally, RA was seropositive in 13 cases and seronegative for both rheumatoid factor and anti-CCP in one case.

**TABLE 1:** Clinical characteristics of 15 patients with γδT-LGL leukemia and rheumatic disease.

Patient no./Sex/Age (y)^a^	RD	^a^Duration of RD (y)	Spleno-megaly	RF/anti-CCP	Absolute leukocyte count (×10^9^/L)	Absolute neutrophil count (×10^9^/L)	Absolute lymphocyte count (×10^9^/L)	Absolute LGL count (×10^9^/L)	Percentage of lymphocytes in the BM
1. M/52	RA	15	+	+/+	1.100	0.055	0.770	0.297	20.2
2. F/55	RA SS	7	−	+/+	2.400	0.840	0.912	0.720	ND
3. F/52	RA SS	7	−	+/+	5.600	1.400	2.816	1.920	ND
4. M/54	RA	1	−	+/+	7.700	1.463	5.852	5.467	35.2
5. F/64	RA	34	−	+/+	3.900	1.287	2.028	0.858	16.4
6. F/67	RA	0	+	+/+	2.800	1.428	1.148	0.126	8.4
7. F/59	RA	7	−	−/−	10.200	3.468	5.916	4.182	15.8
8. F/68	RA	8	+	+/+	1.970	0.177	1.281	ND	23.2
9. M/42	RA	0	+	+/+	1.900	0.551	0.931	ND	7.8
10. M/76	RA	0	+	+/+	2.700	0.066	1.936	0.660	16.2
11. F/58	RA	31	+	+/+	0.700	0.112	0.476	0.063	18.4
12. M/62	RA	T-LGLL → RA/3	+	+/−	2.500	0.100	2.075	0.850	71.6
13. F/60	RA	18	−	+/+	3.500	0.035	2.415	1.330	31.6
14. M/39	RA	0	+	+/+	2.800	0.252	2.044	0.784	ND
15. F/40	CAPS	0	+	ND	7.600	0.684	6.232	4.940	ND

^a^ , at the time of detection neutropenia, lymphocytosis or splenomegaly; y, years; RF, rheumatoid factor; anti-CCP, antibodies against cyclic citrullinated peptides; BM, bone marrow; PB, peripheral blood; +, positive/present; −, negative/absent; ND, no data; LGLs, large granular lymphocytes; RD, rheumatic disease; RA, rheumatoid arthritis; SS, Sjögren syndrome; CAPS, catastrophic antiphospholipid syndrome; T-LGLL, T-cell large granular lymphocytic leukemia.

In five cases, rheumatologic disease was diagnosed simultaneously with the manifestation of T-LGL leukemia, and in one patient, T-LGL leukemia manifested 3 years before the clinical manifestations of RA. The other nine patients developed T-LGL leukemia between 1 and 34 years (median 15 years) after RA diagnosis.

Neutropenia (<1.5 × 10^9^/L) was detected in 14 (93%) cases; the median absolute neutrophil count was 0.551 × 10^9^/L (range, 0.035–3.468 ×10^9^/L). The median lymphocyte count was 2.028 × 10^9^/L (range, 0.476–6.232 × 10^9^/L). Lymphocytosis over 5.000 × 10^9^/L was diagnosed in three patients. The absolute LGL count was known in 13 patients and the median LGL count was 0.850 (range, 0.063–5.467 × 10^9^/L). Absolute LGL count in peripheral blood was more than 2 × 10^9^/L in three cases, less than 2 × 10^9^/L but more than 0.5 × 10^9^/L in seven patients, and less than 0.5 × 10^9^/L in three cases. Splenomegaly was observed in nine (60%) patients and splenectomy was performed in four patients for therapeutic or diagnostic purposes.

**FIGURE 1:**
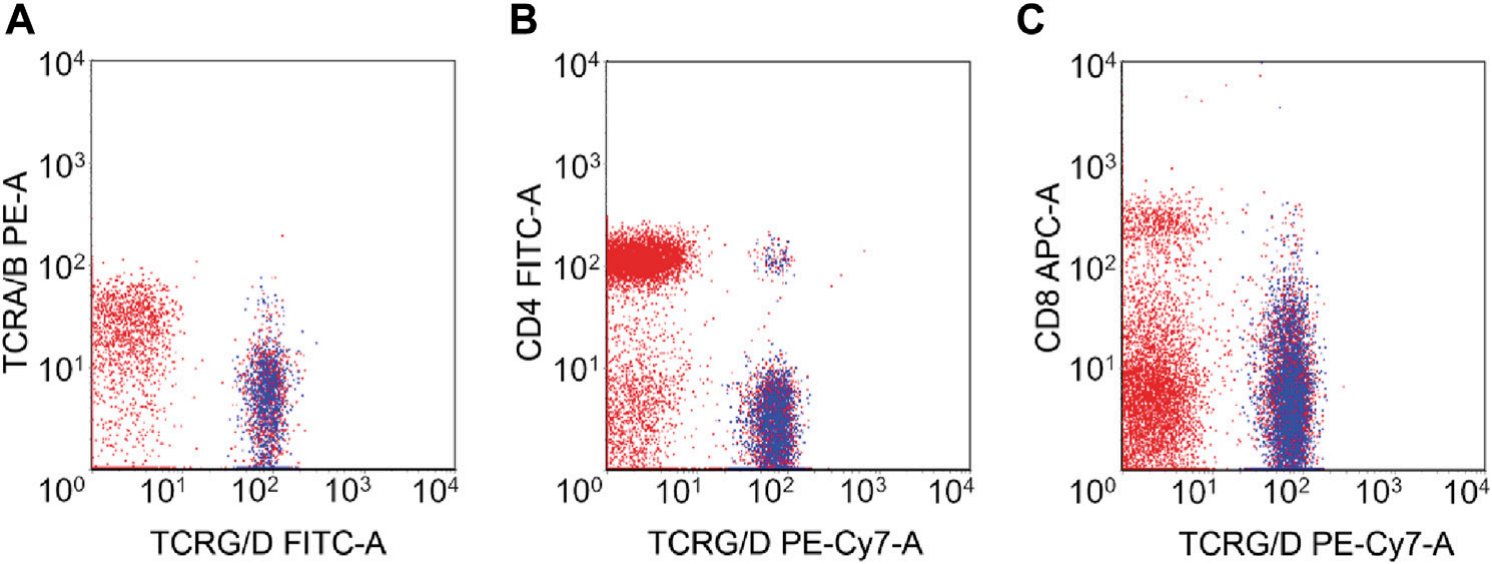
Flow cytometry of γδT-LGL leukemia with CD4−/CD8− phenotype. The neoplastic cells (highlighted in blue) expressed TCRγδ **(A)** but lacked the expression of CD4 **(B)** and CD8 **(C)**.

Immunophenotypic characteristics of γδT-LGL leukemia are presented in Table 2. CD4^−^/CD8^−^ (Figure 1) and CD4^−^/CD8^+^ subtype γδT-LGL leukemia were detected in seven (50%) cases each. For one case, information on CD8 expression on tumor cells was not available. CD5, CD16, CD56, and CD57 were detected on tumor cells in γδT-LGL leukemia in 43% (6/14), 50% (7/14), 8% (1/13), and 64% (9/14) of cases, respectively.

**TABLE 2:** Immunophenotype of 15 patients with γδT-LGL leukemia and rheumatic disease.

Patient no.	Samples for testing/evaluation method	CD3	CD4	CD8	CD5	CD16	CD56	CD57
1	BM/FC and IHC	+	−	ND	ND	ND	ND	+
2	PB/FC	+	−	+	+	+	−	+
3	PB/FC	+	−	+	−	−	−	+
4	PB/FC	+	−	+	−	−	−	−
5	PB/FC	+	−	+	−	−	−	+
6	PB/FC	+	−	−	−	−	ND	ND
7	PB/FC	+	−	+	+	+	+	+
8	Spleen/IHC	+	−	−	−	+	−	−
9	Spleen/IHC	+	−	−	−	+	−	−
10	BM/FC and Spleen/IHC	+	−	−	−	−	−	−
11	Spleen/IHC and FC	+	−	−	−	+	−	−
12	PB/FC	+	−	−	+	−	−	+
13	PB/FC	+	−	−	+	−	−	+
14	PB/FC	+	−	+	+	+	−	+
15	PB/FC BM/IHC	+	−	+	−	+	−	+

FC, flow cytometric analysis; IHC, immunohistochemistry analysis; ND, no data; BM, bone marrow; PB, peripheral blood; +, positive/present; −, negative/absent.

*TCR* monoclonal rearrangement was detected in all 15 patients in at least one of the samples tested (blood, bone marrow, or spleen) (Table 3). Bi-allelic recombination was found in 18 samples with clonal *TCR-δ* rearrangement and one sample showed monoallelic recombination. In samples with a monoclonal *TCR-δ* rearrangement, *TCR-γ* rearrangement was monoclonal in 17 samples and polyclonal in two samples. For *TCR-β,* polyclonal rearrangement was detected in 11 samples, incomplete monoclonal rearrangement in six samples, and only two samples showed complete monoclonal rearrangement.

**TABLE 3:** Molecular characteristics of 15 patients with γδT-LGL leukemia and rheumatic disease.

Patient no.	Samples for testing	Percentage of γδT cells by FC	*TCR* rearrangement			*STAT3* mutation (VAF %)	*STAT5B* mutation (VAF %)
			Gamma	Beta	Delta		
1	PB	2.7	Poly	Poly	Poly	−	−
	BM	18.8	Mono	Mono (C)	Mono	D661Y (2.8%)	−
2	PB	7.3	Mono	Poly	Mono	−	−
3	PB	38.7	Mono	Poly	Mono	D661Y (9.6%)	−
4	PB	59.6	Mono	Poly	Mono	Y640F (18.8%)	−
5	PB	34.4	Mono	Mono (C)	Mono	N647I (1%)	−
6	PB	10.6	Poly	Mono (C) doubtful	Mono	Y640F (1.2%)	−
	BM	ND	Mono doubtful	Mono (C)	Mono	Y640F (0.8%)	−
7	PB	40.0	Mono	Mono (A, B, С)	Mono	−	−
8	Spleen	ND	Mono	Poly	Mono	−	ND
9	PB	ND	Poly	Poly	Poly	−	ND
	BM	2.6	Mono	Poly	Mono	Y640F (0.8%)	−
	Spleen	ND	Mono	Poly	Mono	Y640F (14%)	−
10	PB	ND	Poly	Poly	Poly	−	−
	BM	55.0	Mono	Poly	Mono	S614R (9.2%)	−
	Spleen	ND	Mono	Poly	Mono	S614R (13.8%); N647I (3.5%) Y640F (0.8%)	−
11	PB	3.2	Poly	Poly	Poly	−	−
	BM	ND	Poly	Poly	Mono	S614R (0.8%)	−
	Spleen	37.7	Mono	Poly	Mono	S614R (9.2%)	−
12	PB	27.9	Mono	Mono (B, C)	Mono^#^	S614R (9.6%)	−
13	PB	38.6	Mono	Mono (C)	Mono	Y640F (14.1%)	−
14	PB	39.3	Mono	Poly	Mono	Y640F (9%)	−
15	PB	73.7	Mono	Mono (C)	Mono	D661Y (33.9%)	−

^a^ , at the time of detection neutropenia, lymphocytosis or splenomegaly; y, years; PB, peripheral blood; BM, bone marrow; +, positive/present; −, negative/absent; ND, no data; *STAT*, signal transducer and activator of transcription gene; VAF, variant allele frequency; Mono, monoclonal rearrangement; Poly, polyclonal rearrangement; #, this case had monoallelic *TCR-δ*, recombination.

Point mutations in the *STAT3* gene were identified in at least one of the tested samples in 12 (80%) of 15 examined patients. The following mutations were identified: Y640F (6 cases), D661Y (3 cases), S614R (3 cases), and N647I (2 cases). One patient had multiple *STAT3* gene mutations. *STAT5B* gene mutations were not detected in any of the 14 patients examined.

**FIGURE 2:**
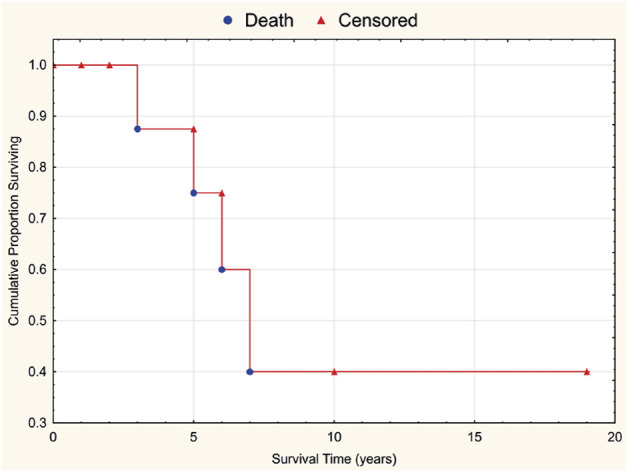
Overall survival curve for 15 patients with γδT-LGL leukemia associated with rheumatic diseases.

A Kaplan–Meier overall survival curve for the 15 patients after γδT-LGL leukemia manifestation is presented in Figure 2. The median follow-up was 3 years (range, 0–19 years). For the four deceased patients the causes of death were hemorrhagic stroke, amyotrophic lateral sclerosis, sepsis, and COVID-19, respectively. The 5-year overall survival was 75%.

## 4 Discussion

γδT-LGL leukemia is a rare and heterogenous pathology, accounting for approximately 17% of all cases of T-LGL leukemia (Bareau et al., 2010; Zhu et al., 2020). A recently published international study involving eight centers over a 23-year period identified only 137 patients with γδT-LGL leukemia (Barilà et al., 2023).

In patients with γδT-LGL leukemia, the prevalence of RA ranges from 16 to 20% (Sandberg et al., 2006; Bourgault-Rouxel et al., 2008) to 43% (Yabe et al., 2015). In the largest cohort of patients with γδT-LGL leukemia reported to date, RA was detected in 12% of patients (16 of 137 patients) (Barilà et al., 2023). In another study, in 11 of 19 patients with RA and T-cell clonality, T-LGL lymphocytes were γδTCR-positive (Schwaneck et al., 2018). In our cohort of γδT-LGL leukemia, all but one patient had RA and only one patient suffered from primary CAPS.

Typically, T-LGL leukemia develops after a long course of RA, suggesting that prolonged immune stimulation is the driver of clonal expansion of cytotoxic T cells (Burks and Loughran, 2006). However, in rare cases, T-LGL leukemia may precede the clinical manifestations of RA by several years or both diseases may manifest simultaneously (Lamy and Loughran, 2003; Shah et al., 2009; Schrenk et al., 2013; Hasanov et al., 2018). Interestingly, in our cohort, in 36% of patients, γδT-LGL leukemia manifested either before or concurrently with RA, suggesting that γδT-LGL leukemia contributes to the pathogenesis of RA. The role of LGL leukemia as a cause of RA in some cases is under debate (Moosic et al., 2022; Marchand and Lamy, 2024). The underlying basis for this causal relationship is the ability of cytotoxic CD8-positive T lymphocytes to produce perforin. Perforin is able to form pores in the target cell membrane, which causes an influx of ions, especially calcium (Law et al., 2010). Increased intracellular calcium leads to conformational changes and hyperactivation of peptidylarginine deiminases, followed by citrullination of proteins with a wide range of molecular masses (hypercitrullination) (Romero et al., 2013). Uncontrolled hypercitrullination generates excessive amounts of antigens that can initiate autoantibody formation and RA in genetically predisposed individuals. In addition, tumor-derived T-LGLs can infiltrate and directly damage various tissues and organs (Rosche et al., 2004; Sarny et al., 2020; Abu Rous et al., 2022; Zhao et al., 2022).

Neutropenia and splenomegaly were the main relevant clinical features in our cohort (observed in 93% and 60% of patients, respectively). However, Barilà et al. reported that the incidence of neutropenia and splenomegaly in γδT-LGL leukemia is 54% and 21%, respectively (Barilà et al., 2023). The higher frequency of these manifestations of γδT-LGL leukemia in our study may be explained by a systematic bias in the selection of patients. Neutropenia and splenomegaly in our patients with seropositive RA were initially misdiagnosed as extra-articular manifestations of RA.

A particular diagnostic challenge is presented by cases with low numbers of LGLs in the peripheral blood. In 62% of patients in our cohort, the absolute number of LGLs in peripheral blood was less than 1.0×10^9^/L. Moreover, in four patients (cases #1 and 9–11), an examination of T-cell clonality by a fragment analysis failed to identify the dominant clone in the peripheral blood and only a bone marrow study revealed the (mono)clonal rearrangement of *TCR* genes. In two of these four cases with a defined percentage of γδT-lymphocytes in the blood, reference values were not exceeded (≤5%).

There are conflicting data on the prevalence of the CD4^−^/CD8^+^ or CD4^−^/CD8^−^ phenotype of γδT-LGL leukemia. Bourgault-Rouxel et al. reported that 13 of 20 cases of γδT-LGL leukemia had a CD4^−^/CD8^+^ phenotype (Bourgault-Rouxel et al., 2008). In the large multicentric study of γδT-LGL leukemia, tumor lymphocytes usually display CD8 positivity (64 of 105 cases), with 23 of 105 cases showing partial CD8 expression (Barilà et al., 2023). Conversely, Yabe et al. observed CD4^−^/CD8^−^ γδT-LGL leukemia in eight out of 12 patients (Yabe et al., 2015). In our cohort, equal numbers of γδT-LGL leukemia cases showed the CD4^−^/CD8^−^ and CD4^−^/CD8^+^ phenotypes. Similarly, equal numbers of patients with the CD4^−^/CD8^−^ and CD4^−^/CD8^+^ phenotypes were found in a small cohort of six patients with γδT-LGL leukemia in a study by Yamane et al. (Yamane et al., 2020). CD16, CD56, and CD57 were expressed in γδT-LGL in our cohort less frequently than in the cohort presented by Barilà et al. (Barilà et al., 2023): 50% vs 72.3%, 8% vs 31.1%, and 64% vs 78.4%, respectively.

Data on the frequencies of *STAT3* and *STAT5B* gene mutations in γδT-LGL leukemia are rare and inconsistent. An analysis of the mutational landscape of γδT-LGL leukemia in six patients by Yamane et al. revealed *STAT3* mutations in all cases and no *STAT5B* mutations (Yamane et al., 2020). In the largest study of γδT-LGL leukemia to date by Barilà et al., mutations in *STAT3* were identified in 37 of 97 patients (38.1%) and mutations in *STAT5B* were detected in four of 94 patients (4.8%) (Barilà et al., 2023). A number of studies have shown that patients with mutations in the *STAT3* gene are more likely to develop RA than patients without these mutations (Jerez et al., 2012; Koskela et al., 2012; Shi et al., 2018). It is possible that the higher frequency of *STAT3* mutations in our study than in Barilà et al. (Barilà et al., 2023) (80% vs 38.1%) can be explained by the presence of RA in the majority of patients in our cohort.

Mutations in the *STAT5B* gene were not detected in our study. It may be related to the relative rarity of mutations in this gene in γδT-LGL leukemia and the mutually exclusive nature of *STAT5B* and *STAT3* variants of T-LGL leukemia (Andersson et al., 2013; Rajala et al., 2013).

Three cases (#9–11) in which T-cell clonality and VAF of *STAT3* mutations were evaluated in blood, bone marrow, and spleen tissues are of particular interest. These patients had a uniform clinical presentation with profound neutropenia, normal or decreased lymphocyte counts in blood, and massive splenomegaly. In all of these cases, the dominant clone was not detected in the peripheral blood, but it was detected in the bone marrow and spleen by fragment analysis. More importantly, *STAT3* mutations were not detected in the peripheral blood, but were detected in the spleen and bone marrow, and VAFs of *STAT3* mutations were significantly higher in the spleen than in the bone marrow.

The spleen is thought to be involved secondary to T-LGL leukemia. However, the findings in these three patients rather suggest the opposite. We speculate that in some patients the spleen is the primary site of tumor growth, with subsequent involvement of the bone marrow and peripheral blood.

Interestingly, the cases with preferential involvement of the spleen had CD4^−^/CD8^−^ phenotype. Five cases with similar clinical parameters were reported by Chen et al. (Chen et al., 2011), including one patient with RA. Another case (#1) presented an identical clinical picture, with tumor involvement of the bone marrow but without peripheral blood involvement (or tumor cell counts in peripheral blood below the detection sensitivity of the methods). This patient also had significant splenomegaly, but splenectomy was not performed.

Distinguishing between γδT-LGL leukemia and hepatosplenic T-cell lymphoma can be challenging, particularly when patients present with marked splenomegaly and no identifiable peripheral blood tumor involvement. Differential diagnosis in these cases is based on the results of comprehensive studies of the bone marrow and the spleen (Chen and Peterson, 2012; Benjamini et al., 2013; Yabe et al., 2017; Shi and Morice, 2024).

## 5 Conclusion

Here, we described clinical and biological features of γδT-LGL leukemia in a cohort of 15 patients with rheumatologic diseases, mostly RA. The frequencies of the CD4^−^/CD8^−^ and CD4^−^/CD8^+^ phenotypes were equal. Mutations in *STAT3* were detected in 80% of cases, while mutations in *STAT5B* gene were not found. Rare cases with a CD4^−^/CD8^−^ phenotype and preferential (compared to bone marrow and peripheral blood) involvement of the spleen were identified. In 36% of cases, γδT-LGL leukemia presented at the same time or earlier than the clinical manifestations of RA, suggesting that γδT-LGL leukemia is a risk factor for the development of RA.

## Data Availability

The data presented in the study are deposited in the SRA repository (https://www.ncbi.nlm.nih.gov/sra), accession number PRJNA1137849.
